# Negative self-schemas drive pathological doubt in OCD

**DOI:** 10.3389/fpsyt.2023.1304061

**Published:** 2023-12-22

**Authors:** Felix Schoeller

**Affiliations:** ^1^Institute for Advanced Consciousness Studies, Santa Monica, CA, United States; ^2^Media Lab, Massachusetts Institute of Technology, Cambridge, MA, United States

**Keywords:** obsessive-compulsive disorder, Bayesian inference, self-model, hyperactive inference, self-confidence, precision, shame, mirroring

## Introduction

Obessive compulsive disorder (OCD) is characterized by distressing, ego-dystonic thoughts (a.k.a., obsessions, O) and repetitive behaviors (a.k.a., compulsions, C) aimed at reducing the anxiety they generate (1). OCD affects 2%−3% of the US population and severely impairs functioning (2). OCD obsessions are characterized by exaggerated doubts disconnected from reality (3). Common themes include contamination (“I may be sick or dirty”), responsibility for harm (“I may harm people or myself”), inability to control thoughts (“I may lose control of my mind”), or being immoral (“I may be a pedophile or evil”). While illogical, doubts persist despite contradictory evidence (4), a source of considerable distress, which clinical diagnosis usually relieves (Table 1). In this short opinion article, we discuss how viewing OCD as pathological self-uncertainty provides novel insights into targeting core cognitive representations for effective treatment. We review converging evidence and provide a unifying framework for understanding OCD that can inform more targeted and effective therapeutic approaches.

**Table 1:** Various types of obsessions and compulsions and their associated phenomenology.

**Obsession**	**Associated phenomenology**	**Compulsion**	**Associated phenomenology**
Hygiene	“I feel contaminated and dirty”	Washing	“I need to wash my hands repeatedly to feel clean”
Sexual	“I have intrusive sexual thoughts that make me feel ashamed”	Checking	“I constantly check if I've done something inappropriate or harmful”
Aggressive	“I fear I might harm myself or others”	Avoiding	“I avoid objects or perform certain actions to prevent harm”
Memory	“I can't stop thinking about the teapot I think I forgot on the stove”	Counting	“If I knock on the teapot five times I'll know for sure it's not on the stove”
Religious	“I am posessed by the devil”	Rituals	“If I don't walk on people's shadows then I won't hurt them”
Somatic	“Something is wrong with my body”	Checking	“I need to take full breath to make sure I don't have dyspnea”

## A dysfunctional self-model drives OCD

**Figure 1:**
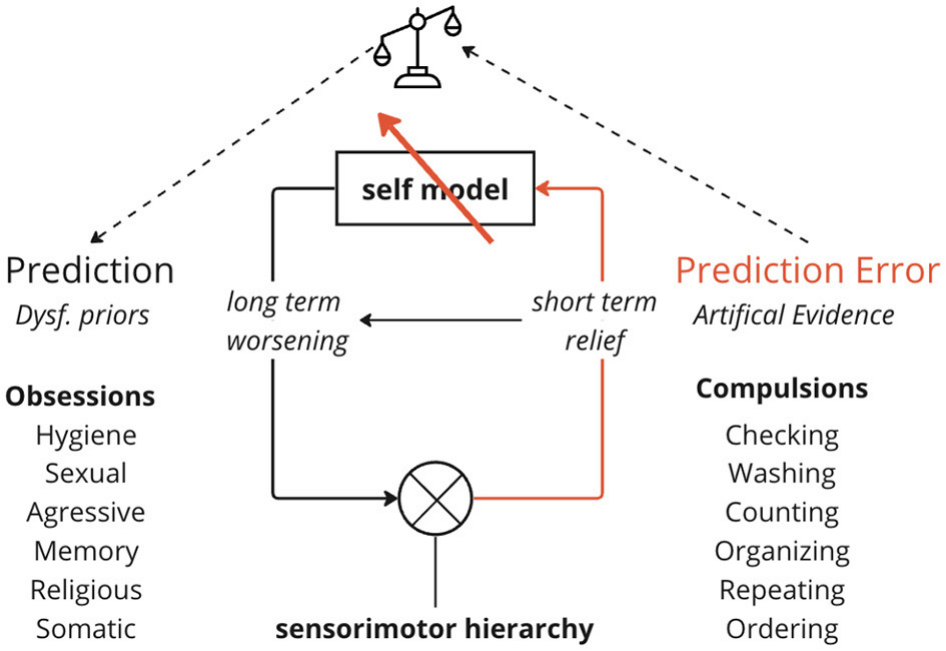
The self-reinforcing cycle in OCD. A dysfunctional negative self-model generates obsessive doubts and anxiety. Compulsive rituals provide temporary relief of anxiety but ultimately reinforce the negative self-model. Arrows indicate the downward flow of obsessive predictions based on the faulty self-model, and the upward flow of “evidence” from compulsions attempting to disconfirm those predictions. This short-term relief from compulsions perpetuates the cycle long-term, as core beliefs are not addressed. Effective treatment must target the dysfunctional self-representations at the root rather than just surface compulsions.

Recent evidence suggest a model of OCD as stemming from flawed high-level priors contained in a dysfunctional self-model (5–13). Here, compulsions are conceptualized as artificially generated evidence to resolve higher order self-uncertainty (Figure 1). The anxiety from negative self-models signifies lost confidence in internal representations and obsessions are a threat to a minimally precise self-concept (9, 14, 15). All things being equal, an uncertain self-model would inevitably skew toward more negative content, since negative experiences are more potent (16) and more difficult to move past than positive ones (17). Deep brain stimulation to the ventral anterior limb of the internal capsule (vALIC), a region associated with self-confidence, rapidly reduced OCD symptoms (18), suggesting this area is critically involved in uncertainty processes that underlie the disorder. Interventions specifically aimed at changing self-perceptions improve cognitive-behavioral treatments for OCD (5, 6, 19). Obsessions likely arise from high-level models encoding dysfunctional assumptions about the self as unreliable or dangerous—a.k.a., the feared self (5, 6, 9, 20). In technical term this amounts to low precision or reliability, a common risk factor for psychopathology (21, 22). The fact that OCD patients feel compelled to perform rituals to reduce anxiety, rather than simply consciously dismissing the obsessional thoughts, suggests that the dysfunctional priors generating the predictions are inaccessible to consciousness. Lacking evidence, OCD patients compulsively create *artifical evidence* for relief (23).

## Hyperactive inference perpetuates pathology

This process is perhaps best captured by the mechanics of active inference—the idea that the brain continuously generates and updates a model of the world using probabilistic, predictive processing (24–26). Active inference involves combining prior beliefs (existing assumptions) with sensory evidence to produce posterior beliefs (updated assumptions) via Bayesian inference (see Figure 1). In OCD, this process becomes pathological when patients assign excessive precision (certainty) to distorted prior beliefs about the self as unpredictable (unreliable, dangerous). In extreme cases, this may lead to the fear of self (6, 27, 28). Self-uncertainty in this context leads to increased volatility in the self-model, opening up the range of more extreme positive or negative beliefs about the self. This produces an overabundance of anxiety-provoking prediction errors signaling mismatch between expectations and observations (29). To reduce this anxiety, patients compulsively engage in exhaustive Bayesian updating, relying on compulsive behaviors to generate new, artificial sensory evidence (observable outcomes of handwashing, counting, checking, etc). However, compulsions cannot provide absolute certainty about the abstract self, fueling renewed doubt. Thus, the relief is only temporary, and in the long term reinforces the negative self-model that something must be wrong with the patient (Figure 1). This is akin to a man trying to bail water out of a ship with a deep rupture in its hull. Each bucket generates temporary relief (“I would not be O if I did C”) but the leak is not being addressed (leading to “D”). Targeting core self-models, not just surface symptoms, is therefore crucial (30, 31).

## The roots of self-uncertainty

Changing flawed assumptions about the self, and addressing the self-uncertainty driving pathological doubt may enable lasting improvement and eliminate the compulsive need for constant testing and reassurance (5, 6, 19, 32, 33). But what influences the valence of the self-model in the first place such that it attempts to generate artificial prediction errors to disprove it? Developmental psychology emphasizes that the quality of early bonding with primary caregivers shapes one's internal working models about the self and relationships. Dysfunctional behavioral patterns resulting from suboptimal caregiver interactions can lead to an unstable sense of self (34–36). Ultimately, insecure attachment patterns lead to doubts about one's worth and lovability (37, 38) and the formation of OCD symptoms (39). From the infant's perspective, maternal mirroring provides essential external validation for consolidating a coherent self-concept (40–42). Parents who feel opposed, rejected, and incapable tend to produce children who undervalue themselves (43). As the mother mimics the baby's facial expressions and reflects their emotions—e.g., through exaggerated infant-directed speech (a.k.a., motherese)—the infant learns to associate their inner experience with the mother's attuned external responses (44, 45). Among others, this allows the infant to gain self-awareness, in the process of seing their inner experience mirrored externally (46). This process of mirroring—a.k.a., reflective functioning (47) or attunement (42)—establishes the foundations for secure attachment (35), ultimately allowing mentalization and introspective abilities to unfold (48). Through repeated, predictable mirroring, the infant comes to understand their needs and develop trust that caregivers will be present to attend them and help regulate arousal. The exaggerated self-focus and hyperactive inference characteristic of OCD points to lack of proper mirroring from caregivers to help build realistic, balanced self-representations (40). Early conditioning may explain the development of rigid, deeply engrained negative self-schemas in OCD, often leading to difficulty in engaging in self-reinforcing social rituals (e.g., birthdays, graduations, and weddings). Recent clinical trials show psychoplastogenic drugs like psilocybin can rapidly reduce OCD symptoms, potentially by “resetting” these maladaptive self-models through heightened neuroplasticity (49).

## Conclusion

A model of OCD as dysfunctional high-level priors and hyperactive inference driven by a negative, uncertain self-model provides insight into the disorder. It leads to the prediction that compulsive rituals should continue as long as self-uncertainty and flawed negative self-priors remain unadressed. We suggested the term hyperactive inference to explain compulsions as excessive Bayesian updating driven by inaccurate priors and a futile attempt to alleviate uncertainty about the self. The temporary relief of compulsive rituals ultimately reinforces the negative schemas generating pathological doubt and anxiety. Effective treatment should target core cognitive representations of the self rather than just surface symptoms. While the obsessional thoughts arise internally, the outer environment is more or less conducive to carrying out the compulsive rituals, with certain settings and situations providing more opportunity to engage in behaviors like checking, washing, counting, or arranging. Further research should explore neural implementations of this framework and implications for therapeutic approaches focused on the roots of uncertainty underlying OCD. By better understanding the wellspring of pathological inference in OCD, more targeted and effective interventions can be developed, modulating the self-confidence and deeply engrained assumptions at the origins of perfectionism and self-monitoring.

## Author contributions

FS: Writing—original draft.
